# Synthesis and *in vitro* Antitumor Potency of (Cyclohexane-1,2-Diamine)Platinum(II) Complexes with Aminotris(Methylenephosphonic Acid) as Bone-Seeking Ligand

**DOI:** 10.1155/BCA.2005.179

**Published:** 2005

**Authors:** Markus Galanski, Susanna Slaby, Michael A. Jakupec, Bernhard K. Keppler

**Affiliations:** Institute of Inorganic Chemistry University of Vienna Waehringerstr. 42 Vienna A-1090 Austria

**Keywords:** platinum complexes, anticancer activity, cytotoxicity, osteotropic phosphonates

## Abstract

In order to develop platinum complexes with selective activity in primary and secondary bone malignancies and with the aim to optimize antitumor activity, platinum(II) complexes with aminotris(methylenephosphonic acid) as bone-seeking (osteotropic) ligand have been synthesized, characterized and tested in the cisplatin-sensitive ovarian carcinoma cell line CH1. As non-leaving diamine ligands, which are decisive for the cellular processing of DNA adducts, *cis-R,S*-cyclohexane-1,2-diamine, *trans-S,S*-cyclohexane-1,2-diamine and *trans-R,R*-cyclohexane-1,2-diamine have been used, resulting in complexes 1, 2, and 3, respectively. The cytotoxicity of the complexes under investigation decreases in the order 3 > 2 > 1 which is in accord with structure-activity relationships with other (cyclohexane-1,2- diamine)platinum(II) and platinum(IV) complexes: Both *trans* complexes (2 and 3) display a higher *in vitro* potency than the corresponding *cis* isomer (I), with the *trans-R,R* isomer (3) being the most active in this series. In comparison to the analogous (cyclohexane-1,2-diamine)platinum(II) complexes with bis(phosphonomethyl)aminoacetic acid as osteotropic carrier ligand, the cytotoxicity of 1-3 was found to be 1.5 – 2 fold higher, which is explainable by a different coordination mode of the phosphonic acid ligands (acetato versus phosphonato).


### Synthesis and *in vitro* Antitumor Potency of (Cyclohexane-1,2-Diamine)Platinum(II) Complexes with Aminotris(Methylenephosphonic Acid) as Bone-Seeking Ligand

Markus Galanski,* Susanna Slaby, Michael A. Jakupec, and Bernhard K. Keppler

*Institute of Inorganic Chemistry, University of Vienna, Waehringerstr. 42, A-1090 Vienna, Austria*

### ABSTRACT

In order to develop platinum complexes with selective activity in primary and secondary bone malignancies and with the aim to optimize antitumor activity, platinum(II) complexes with aminotris(methylenephosphonic acid) as bone-seeking (osteotropic) ligand have been synthesized, characterized and tested in the cisplatin-sensitive ovarian carcinoma cell line CH1. As non-leaving diamine ligands, which are decisive for the cellular processing of DNA adducts, *cis-R,S*-cyclohexane-1,2-diamine, *trans-S,S*-cyclohexane-1,2-diamine and *trans-R,R*-cyclohexane-1,2-diamine have been used, resulting in complexes **1**, **2**, and **3**, respectively. The cytotoxicity of the complexes under investigation decreases in the order **3** > **2** > **1** which is in accord with structure-activity relationships with other (cyclohexane-1,2-diamine)platinum(II) and platinum(IV) complexes: Both *trans* complexes (**2** and **3**) display a higher *in vitro* potency than the corresponding *cis* isomer (**1**), with the *trans-R,R* isomer (**3**) being the most active in this series. In comparison to the analogous (cyclohexane-1,2-diamine)platinum(II) complexes with bis(phosphonomethyl)aminoacetic acid as osteotropic carrier ligand, the cytotoxicity of **1-3** was found to be 1.5 – 2 fold higher, which is explainable by a different coordination mode of the phosphonic acid ligands (acetato versus phosphonato).

**Keywords:** platinum complexes, anticancer activity, cytotoxicity, osteotropic phosphonates

### Abbreviations

ATMP, aminotris(methylenephosphonic acid)

BPMAA, bis(phosphonomethyl)aminoacetic acid

* All correspondence should be addressed to:

tel.: +43-1-4277-52603, Fax: +43-1-4277-52680, e-mail: markus.galanski@univie.ac.at

### INTRODUCTION

Since the recognition of the cytotoxic activity of cisplatin (Figure 1) in the late 1960s /1,2,3,4/, thousands of platinum complexes have been synthesized in order to develop similar drugs with improved antitumor activity and with a better toxicological profile /5,6/. Carboplatin is the result of such attempts; it has, due to the same diammineplatinum(II) moiety, equivalent efficacy but milder toxicity because of the 1,1-cyclobutanedicarboxylato leaving group.

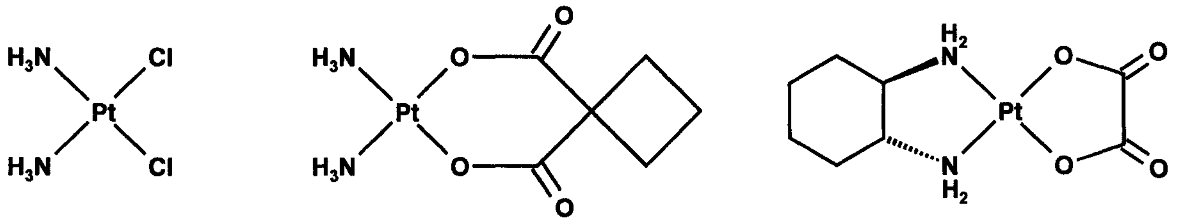

**Fig. 1:** Platinum(II) complexes in worldwide clinical use: cisplatin (left), carboplatin (middle) and oxaliplatin (right).

In the case of oxaliplatin, which has been approved in 1999 in Europe and in 2002 in the US, the substitution of the two ammine ligands with the cyclohexane-1,2-diamine (or DACH = diaminocyclohexane) moiety led to the third platinum-based anticancer compound in worldwide clinical use with good antitumor properties and partial lack of cross-resistance with cisplatin /7,8/. Oxaliplatin (Eloxatin; Sanofi-Synthelabo), which has a more favorable safety profile than cisplatin, is indicated in combination with 5-fluorouracil (5-FU) and leucovorin for patients with colorectal cancer.

In order to synthesize platinum complexes with selective activity in primary and secondary bone malignancies such as osteosarcoma (bone tumors) as well as in lethal ossifying lung metastases and bone metastases from tumors with other primary sites, a series of osteotropic (bone-seeking) [(bis(phosphonomethyl)amino-κ*N*)acetato-κ*O*(2-)]platinum(II) complexes has been synthesized /9,10,11/.

With the aim of exploring structure-activity relationships, different kinds of diam(m)ine ligands, which are decisive for the cellular processing of DNA adducts, have been used (Figure 2) /12/. However, the bis(phosphonomethyl)-substituted amino acetic acid (BPMAA) ligand, which has a high affinity for calcium and calcified tissues and which is therefore responsible for the carrier-mediated transport to the mineral bone matrix, was left unchanged in this study.

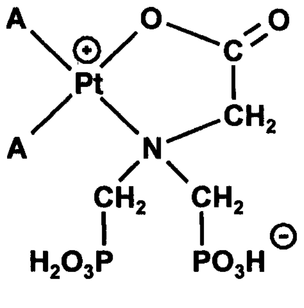

**Fig. 2:** Cytotoxic [(bis(phosphonomethyl)amino-κ*N*)acetato-κ*O*(2-)]platinum(II) complexes displaying osteotropic properties, $A_2$ = diammine, ethane-1,2-diamine, *cis-R,S*-cyclohexane-1,2-diamine, *trans-S,S*-cyclohexane-1,2-diamine or *trans-R,R*-cyclohexane-1,2-diamine.

Within the series of complexes, the *in vitro* antitumor activity in the cisplatin-sensitive ovarian carcinoma cell line CH1 decreases depending on the coordinated diam(m)ine ligand in the following order (Table 1): *trans-R,R*-cyclohexane-1,2-diamine > *trans-S,S*-cyclohexane-1,2-diamine > diammine ≥ *cis-R,S*-cyclohexane-1,2-diamine > ethane-1,2-diamine.

**Table 1**

Cytotoxic activity of [(bis(phosphonomethyl)amino-κ*N*)acetato-κ*O*(2-)]platinum(II) complexes in the ovarian cancer cell line CH1 ($IC_{50}$ values in μM, exposure for 96 hours, MTT assay).

| amine ligand ($A_2$) | $IC_{50}$ value [μM] |
|---|---|
| *trans-R,R*-cyclohexane-1,2-diamine | 20.6 |
| *trans-S,S*-cyclohexane-1,2-diamine | 52.4 |
| diammine | 128 |
| *cis*-R,S-cyclohexane-1,2-diamine | 169 |
| ethane-1,2-diamine | 532 |

In order to set up structure-activity relationships and to optimize antitumor activity of osteotropic platinum-based complexes, we have focused on the synthesis of platinum(II) compounds with (cyclohexane-1,2-diamine) as non-leaving diamine ligand. As bone-seeking carrier ligand a tris(phosphonic acid), ATMP, aminotris(methylenephosphonic acid), was selected.

### 2. EXPERIMENTAL

The structure of the phosphonatoplatinum(II) complexes under investigation as well as the numbering scheme for the NMR study is illustrated in Figure 3. We will refer to these compounds as complexes **1**, **2** and

3 in case of a *cis-R,S-*, *trans-S,S-* and *trans-R,R*-cyclohexane-1,2-diamine ligand, respectively.

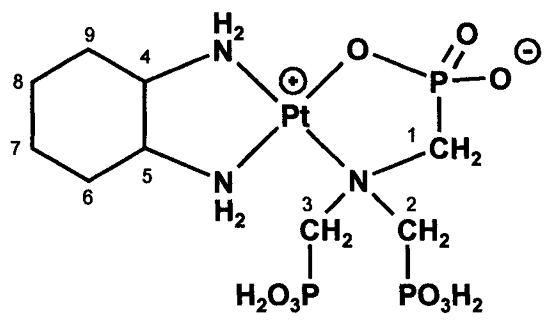

**Fig. 3:** Structure and NMR numbering scheme of the phosphonatoplatinum(II) complexes under investigation.

### Chemicals and Supplies for Synthesis

Potassium tetrachloroplatinate(II) was obtained from Degussa. The phosphonic acid aminotris(methylenephosphonic acid), ATMP, was kindly provided by Henkel KGa Düsseldorf. All other chemicals obtained from commercial suppliers were used as received and were of analytical grade. Water was used bidistilled. The synthetic procedures were carried out in a light protected environment.

### NMR Measurements and Elemental Analyses

$^{1}$H, $^{13}$C{$^{1}$H}, $^{31}$P, $^{31}$P{$^{1}$H }, $^{1}$H,$^{1}$H-COSY and $^{13}$C,$^{1}$H,-COSY spectra were recorded in $D_2O$ or $H_2O/D_2O$ (9:1) at 298 K using a Bruker Avance DPX 400 instrument (UltraShield$^{TM}$ Magnet) and standard pulse programmes at 400.13 ($^{1}$H), 100.62 ($^{13}$C) and 162.0 MHz ($^{31}$P). Chemical shifts were measured relative to the solvent peak or to external 85% $H_3PO_4$. Elemental analyses were performed by the microanalytical laboratory at the University of Vienna.

### Syntheses

(*SP*-4-2)-dichloro(*cis-R,S*-cyclohexane-1,2-diamine)platinum(II), (*SP*-4-2)-dichloro(*trans-S,S*-cyclohexane-1,2-diamine)platinum(II) and (*SP*-4-2)-dichloro(*trans-R,R*-cyclohexane-1,2-diamine)platinum(II) have been synthesized according to standard literature procedures starting from $K_2PtCl_4$ /12/.

(*SP*-4-3)/(*SP*-4-4)-[(Bis(phosphonomethyl)amino-κ*N*)methylphosphonato-κ*O*(2-)](*cis*-cyclohexane-1,2-diamine-κ$^{2}$*N,N'*)platinum(II) (**1**)

(*SP*-4-2)-Dichloro(*cis-R,S*-cyclohexane-1,2-diamine)platinum(II) (400 mg, 1.05 mmol) was suspended in 15 ml of water. After addition of silver nitrate (340 mg, 2.0 mmol), the mixture was stirred overnight at room temperature. Silver chloride precipitated and was filtered off. ATMP (299 mg, 1.0 mmol) was added to the yellow solution. After the mixture was stirred for 60 minutes at 50°C and over night at room temperature, the solvent was removed under reduced pressure. The resulting solid was dissolved in water. The phosphonato complex **1** was precipitated with acetone, filtered and dried over $P_2O_5$ under reduced pressure to obtain 509 mg of a white solid; yield 84%. Elemental analysis, found: C, 17.87; H, 3.83; N, 6.71. Calcd for $C_4H_{15}N_3O_8P_2Pt$: C, 17.83; H, 3.99; N, 6.93. $^1H$ NMR in $D_2O$: δ = 1.17 [m, 1H, H(7) or H(8)], 1.31 [m, 2H, H(7) or H(8)], 1.57 [m, 1H, H(7) or H(8)], 1.66 [m, 4H, H(6), H(9)], 2.66 [m, 1H, H(4) or H(5)], 2.91 [m, 1H, H(4) or H(5)], 3.3 - 3.7 [m, 5H, H(1), H(2), H(3)], 3.90 [m, 1H, H(1)], 4.94 [m, 1H, H($N_a$)], 5.43 [m, 1H, H($N_a$)], 5.92 [m, 1H, H($N_b$)], 6.22 [m, 1H, H($N_b$)]. $^{13}C\{^1H\}$ NMR in $D_2O$: δ = 19.4 [C(7) or C(8)], 21.8 [C(7) or C(8)], 25.7 [C(6) or C(9)], 26.1 [C(6) or C(9)], 57.5 [C(4) or C(5)], 59.1 [C(4) or C(5)], 59.2 [d, $^1J_{C,P}$ = 147 Hz, C(1)], 62.4 [d, $^1J_{C,P}$ = 143 Hz, C(2) or C(3)], 63.2 [d, $^1J_{C,P}$ = 140 Hz, C(2) or C(3)]. $^{31}P\{^1H\}$ NMR in $D_2O$: δ =13.3 [2P, $PO_3H_2$], 43.7 [1P, $PO_3Pt$].

(SP-4-2)-[(Bis(phosphonomethyl)amino-κ*N*)methylphosphonato-κ*O*(2-)](*trans-S,S*-cyclohexane-1,2-diamine-κ$^2$*N*,*N'*)platinum(II) (**2**)

The synthetic procedure is the same as that for complex **1**. Yield 76%. Elemental analysis, found: C, 20.07; H, 4.02; N, 6.34. Calcd for $C_4H_{15}N_3O_8P_2Pt{\cdot}0.5\ C_3H_6O$: C, 19.85; H, 4.28; N, 6.61. $^1H$ NMR-Spectrum in $D_2O$: δ = 1.03 [m, 2H, H(7) or H(8)], 1.15 [m, 2H, H(6), H(9)], 1.44 [m, 2H, H(7) or H(8)], 1.93 [m, 2H, H(6), H(9)], 2.27 [m, 2H, H(4), H(5)], 3.35 - 3.75 [m, 5H, H(1),H(2), H(3)], 3.87 [m, 1H, H(1)], 4.90 [m, 1H, H($N_a$)], 5.52 [m, 2H, H($N_a$), H($N_b$)], 6.41 [m, 1H, H($N_b$)]. $^{13}C\{^1H\}$ NMR-Spectrum in $D_2O$: δ = 24.1 [C(7), C(8)], 32.0 [C(6) or C(9)], 32.5 [C(6) or C(9)], 59.2 [d, $^1J_{C,P}$ = 147 Hz, C(1)], 62.1 [C(4) or C(5)], 62.5 [d, $^1J_{C,P}$ = 140 Hz, C(2) or C(3)], 62.8 [C(4) or C(5)], 63.5 [d, $^1J_{C,P}$ = 140 Hz, C(2) or C(3)]. $^{31}P$ NMR-Spectrum in $D_2O$: δ =12.7 [dd, $^2J_{P,H}$ = 11 Hz, 1P, $PO_3H_2$], 13.3 [dd, $^2J_{P,H}$ = 13 Hz 1P, $PO_3H_2$], 44.0 [dd, 1P, $^2J_{P,H}$ = 10 Hz, $PO_3Pt$)].

(*SP*-4-2)-[(Bis(phosphonomethyl)amino-κ*N*)methylphosphonato-κ*O*(2-)](*trans-R,R*-cyclohexane-1,2-diamine-κ$^2$*N*,*N'*)platinum(II) (**3**)

The synthetic procedure is the same as that for complex **1**. Yield 90%. Elemental analysis, found: C, 17.90; H, 3.85; N, 6.70. Calcd for $C_4H_{15}N_3O_8P_2Pt$: C, 17.83; H, 3.99; N, 6.93. $^1H$ NMR in $D_2O$: δ = 1.01 [m, 2H, H(7) or H(8)], 1.14 [m, 2H, H(6), H(9)], 1.43 [m, 2H, H(7) or H(8)], 1.91 [m, 2H, H(6), H(9)], 2.25 [m, 2H, H(4), H(5)], 3.3 - 3.7 [m, 5H, H(1), H(2), H(3)], 3.85 [m, 1H, H(1)], 4.90 [m, 1H, H($N_a$)], 5.51 [m, 2H, H($N_a$), H($N_b$)], 6.37 [m, 1H, H($N_b$)]. $^{31}P\{^1H\}$ NMR in $D_2O$: δ =12.8 [1P, $PO_3H_2$], 13.4 [1P, $PO_3H_2$], 44.0 [1P, $PO_3Pt$].

### Cell Culture and Cytotoxicity Test Conditions

The ovarian carcinoma cell line CH1, which has been established from an ascites sample of a patient with a papillary cystadenocarcinoma of the ovary, was kindly provided by Lloyd R. Kelland (CRC Centre for Cancer Therapeutics, Institute of Cancer Research, Sutton, UK). Cells were grown as adherent monolayer cultures in complete culture medium, i.e., Minimal Essential Medium (MEM) supplemented with 10% heat-inactivated fetal bovine serum, 1 mM sodium pyruvate, 2 mM *L*-glutamine, 50 U/ml penicillin and 50 μg/ml streptomycin (all purchased from Gibco). Cultures were maintained at 37 °C in a humidified atmosphere containing 5% $CO_2$.

Cytotoxicity of complexes **1-3** was determined by means of a colorimetric microculture assay (MTT assay, MTT = 3-(4,5-dimethyl-2-thiazolyl)-2,5-diphenyl-2H-tetrazolium bromide). For this purpose, CH1 cells were harvested from adherent cultures by trypsinization, and suspensions were adjusted to cell densities of $1.25 \times 10^4$ cells/ml in order to assure exponential growth throughout drug exposure. Aliquots of 200 μl/well of these suspensions were used to seed microcultures in 96-well plates. After incubation for 24 hrs, cells were exposed to the test compounds, which were dissolved and serially diluted in complete culture medium shortely before use. Each concentration was given to eight microcultures in parallel. After incubation for four days, drug solutions were removed and replaced by 150 μl/well complete culture medium and 20 μl of aqueous MTT solution (5 mg/ml). After incubation for a further 4 hrs, the medium/MTT mixtures were removed and formazan crystals were dissolved in 150 μl of DMSO/well. Optical densities at 550 nm were measured with a microplate reader (Tecan Spectra Classic), and the quantity of living cells was expressed as T/C values by comparison to untreated control microcultures. The concentrations of complexes that decreased absorption by 50% were calculated by interpolation and taken as the $IC_{50}$ values. Evaluation is based on means of values obtained from three independent experiments.

### 3. RESULTS AND DISCUSSION

### 3.1. Syntheses

Synthesis of the bis(phosphonomethyl)amino-κ*N*)methylphosphonato-κ*O*(2-)](cyclohexane-1,2-diamine-κ$^2$*N*,*N*')platinum(II) complexes **1-3** was accomplished in three steps in analogy to the synthetic procedure of the bis(phosphonomethyl)-substituted aminoacetatoplatinum(II) counterparts, as described previously /12/. The dichloro (cyclohexane-1,2-diamine) platinum(II) complexes are synthesized in one step, starting from $K_2PtCl_4$ and the *cis-R,S-*, *trans-S,S-* and *trans-R,R*-cyclohexane-1,2-diamine ligands. After that, the dichloroplatinum(II) species are activated over night using silver nitrate (Scheme 1).

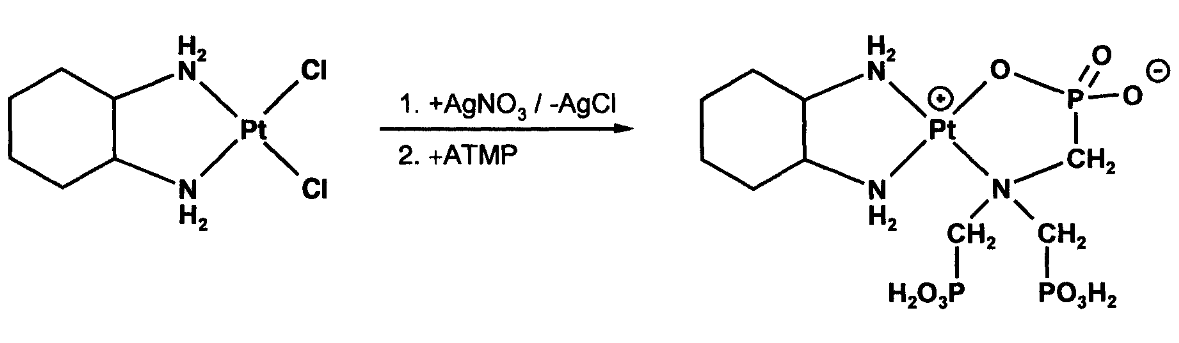

**Scheme 1**

Synthesis of [(bis(phosphonomethyl)amino-κ*N*)methylphosphonato-κ*O*(2-)](cyclohexane-1,2-diamine-κ$^2$*N*,*N*')platinum(II) complexes **1-3**.

After removing the precipitated silver chloride, aminotris(methylenephosphonic acid), ATMP, is added, and the bis(phosphonomethyl)amino-κ*N*)methylphosphonato-κ*O*(2-)]platinum(II) complexes **1-3** are formed.

Purity of the compounds was checked by elemental analysis, whereas coordination of the ATMP ligand can best be judged by $^{31}P$ NMR spectroscopy.

### 3.2. NMR spectroscopy

In phosphorus NMR, the free ligand resonates at 9.3 ppm, whereas in complexes **1-3** a significant downfield shift of the signals can be observed: In the region between 43.7 and 44.0 ppm, a $^{31}P$ signal of the coordinated phosphonato residue is found; resonances of the uncoordinated phosphonomethyl groups are located between 12.7 and 13.4 ppm. In case of the (*trans*-cyclohexane-1,2-diamine)platinum(II) complexes **2** and **3**, two signals can be detected with a separation of 0.6 ppm in the region around 13.0 ppm, reflecting the nonequivalence of the uncoordinated phosphonomethyl groups. This is in accordance with the analogous BPMAA complexes, where signals between 12.6 and 13.0 ppm could be found /12/. In the case of the *cis*-*R*,*S*-cyclohexane-1,2-diamine ligand, two isomers are formed with either $NH_2$-($C^R$) or $NH_2$-($C^S$) in *trans* position to the coordinated phosphonato group. For this mixture, only one unresolved $^{31}P$ chemical shift at 13.3 ppm could be detected.

$^{1}H$ and $^{13}C$ resonance assignment was performed by analysis of two-dimensional $^{1}H$,$^{1}H$ and $^{13}C$,$^{1}H$ shift correlated spectra, as will be demonstrated for complex **1** in the following section. Two kinds of isolated spin systems can be detected in the $^{1}H$,$^{1}H$-COSY NMR spectrum of complex **1** (Figure 4): (i) the protons of the *cis*-cyclohexane-1,2-diamine ligand, which are separated in three regions (1.0-1.8: $CH_2$, 2.5-3.0: CH, 4.8-6.4: $NH_2$) and (ii) the $^{1}H$ resonances of ATMP coordinated to the platinum(II) center (3.3-4.0: $CH_2$). The protons bound to the same nitrogen were detected at 4.94, 5.43 and 5.92, 6.22 ppm, respectively, and can be unequivocally assigned on the basis of their $^{1}H$,$^{1}H$ shift correlation signals.

Starting from these resonances, a vicinal coupling to the methine protons H(4) and H(5) at 2.66 and 2.91 ppm of the cyclohexane ring can be observed. H(4) and H(5) display a distinct cross peak to the neighboring $CH_2$ groups H(6) and H(9) which merge to one unresolved multiplett at 1.66 ppm. Consequently, the resonances at 1.17, 1.31 and 1.57 ppm, which are found in a ratio of 1:2:1, originate from the methylene protons H(7) and H(8).

$^{13}C$ resonance assignment for complex **1** was performed by analyzing $^{13}C$,$^{1}H$ connectivities in the two-dimensional $^{13}C$,$^{1}H$-COSY NMR spectrum (Figure 5).

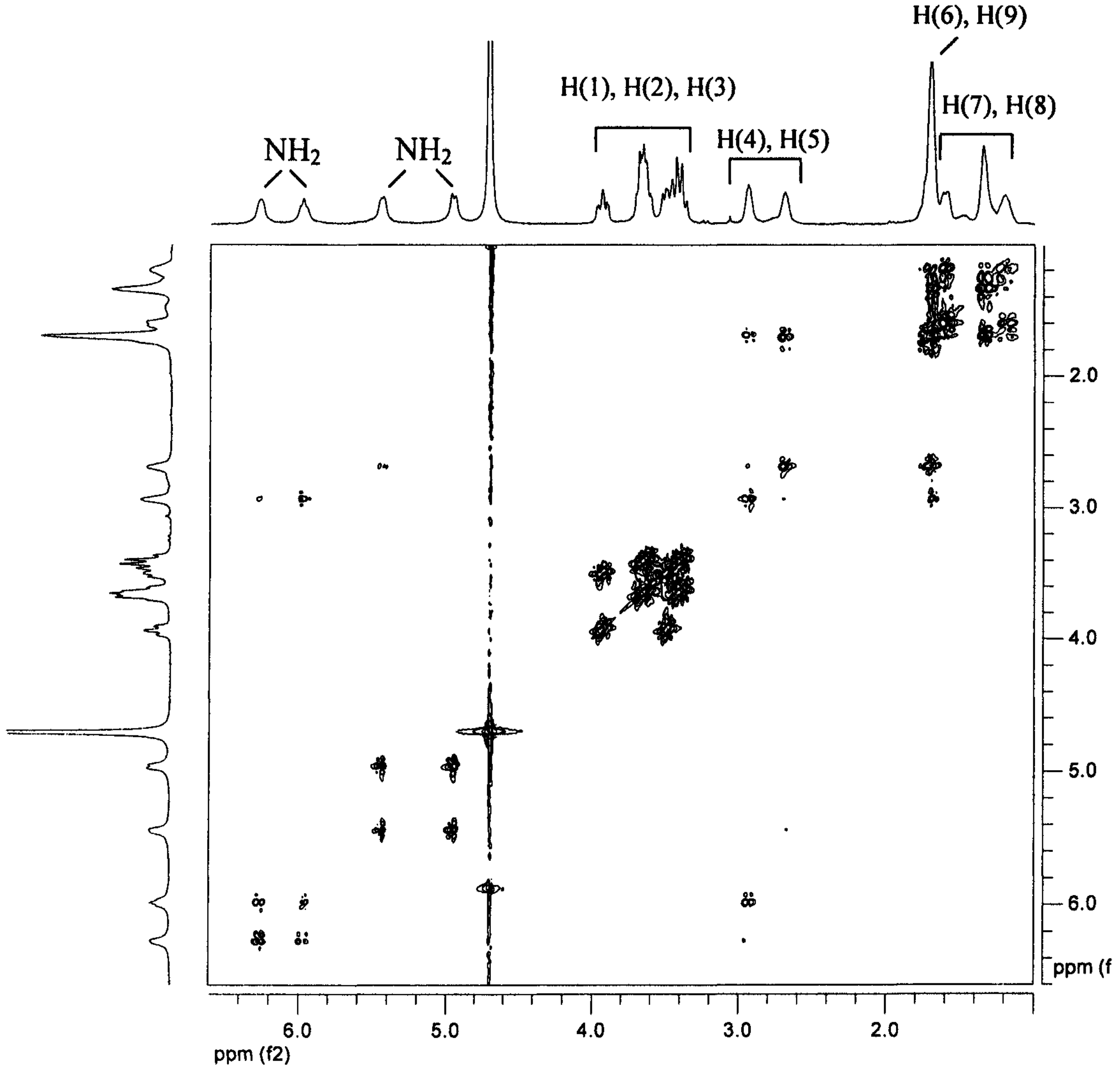

**Fig. 4:** $^{1}H,^{1}H$-COSY NMR spectrum of complex **1** in $D_2O$.

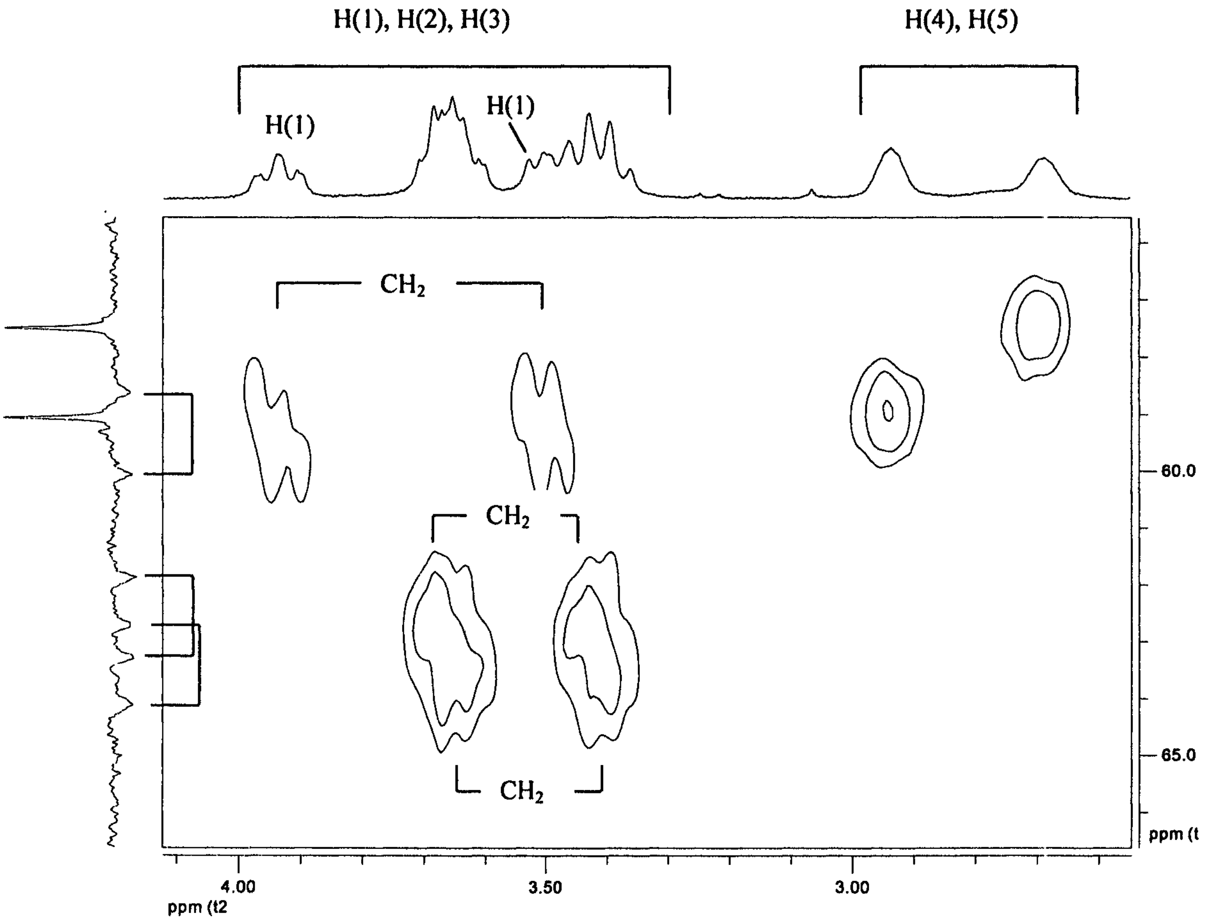

**Fig 5:** $^{13}C,^{1}H$-COSY NMR spectrum of complex **1** in $D_2O$ (region of $^{13}C$ shift correlation signals to protons H(1), H(2), H(3), H(4) and H(5) is shown).

While interpretation of proton and $^{13}C$ resonances of the cyclohexane ring is straightforward, as can be seen in the case of H(4) and H(5), analysis of $^{1}H$ and $^{13}C$ chemical shifts in the coordinated ATMP ligand is more complicated: (i) The protons of each of the three $CH_2$ groups are nonequivalent, and therefore the $^{1}H$ resonances (of protons bound to one carbon atom) are separated, by up to 0.45 ppm, with a severe overlapping of H(2)/H(3) and H(1)/H(2)/H(3) signals. (ii) The protons as well as the $^{13}C$ atoms are coupling with the $^{31}P$ nuclei. (iii) Due to the nonequivalent properties of protons bound to one carbon atom, geminal coupling with $^{2}J_{H,H}$ coupling constants of about 13 Hz is observed. Moreover, these values are in the order of magnitude of $^{2}J_{H,P}$ coupling constants.

The $^{13}C$ resonance of C(1) of the coordinated methylphosphonato moiety was found to be at 59.2 ppm, with a $^{1}J_{C,P}$ coupling of 147 Hz and shift correlation signals to protons H(1). Only one of these $^{1}H$ resonances (at 3.90 ppm) is not superimposed by other proton signals and appears as an isolated triplett due to coupling with the geminal H(1) and the neighboring $^{31}P$ nucleus. $^{13}C$ chemical shifts of the phosphonomethyl groups (C(2)/C(3)) were detected at 62.4 and 63.2 ppm, with $^{1}J_{C,P}$ coupling constants of 147 and 140 Hz, respectively. Shift correlation signals to the corresponding protons (H(2)/H(3)) indicate a separation of the resonances of geminal protons by about 0.2 ppm.

### 3.3. Cytotoxic Activity

The cytotoxic activity of [(bis(phosphonomethyl)amino-κ*N*)methylphosphonato-κ*O*(2-)](cyclohexane-1,2-diamine-$\kappa^2$*N*,*N*')platinum(II) complexes **1-3** has been compared in the highly cisplatin-sensitive human ovarian cancer cell line CH1. Concentration-effect curves were obtained after exposure for 96 hours by means of a colorimetric microculture assay (MTT assay). The concentration-effect curves for **1-3** are shown in Figure 6, whereas the corresponding $IC_{50}$ values are reported in Table 2.

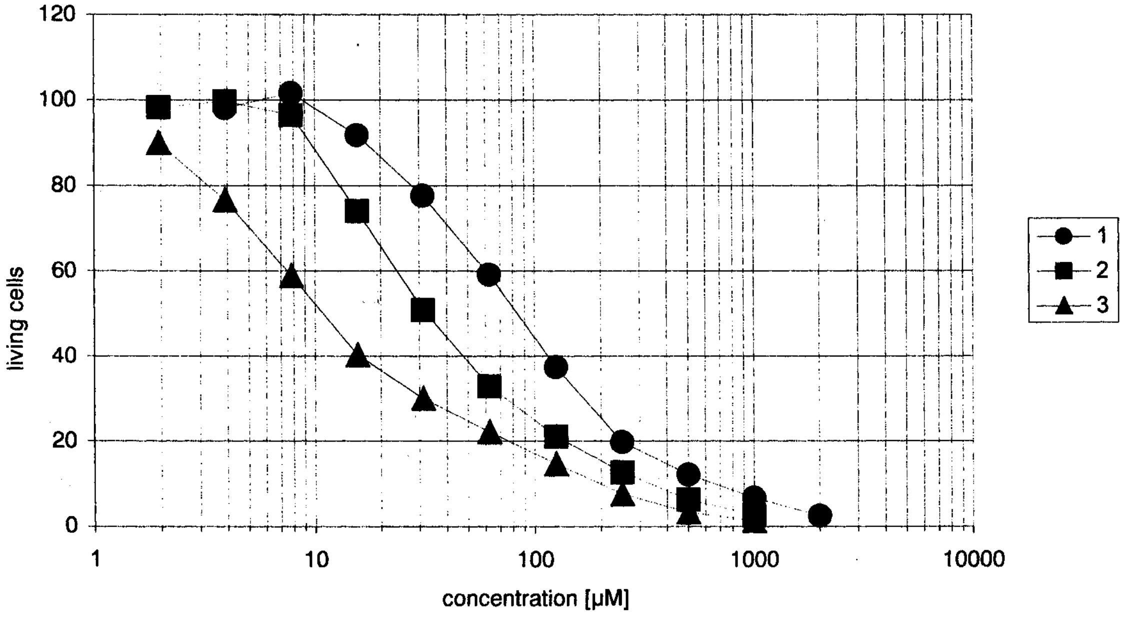

**Fig 6:** Concentration-effect curves of [(bis(phosphonomethyl)amino-κ*N*)methylphosphonato-κ*O*(2-)] (cyclohexane-1,2-diamine-$\kappa^2$*N*,*N*')platinum(II) complexes **1-3** in ovarian cancer cells (CH1) after exposure for 96 hours.

As expected, the *in vitro* antitumor activity decreases depending on the stereochemistry of the cytotoxic (cyclohexane-1,2-diamine)platinum(II) moiety in the following order: **3** > **2** > **1**. Both *trans* complexes **3** and **2** display a higher potency than the corresponding *cis* isomer **1**, with the (*SP*-4-2)-[(bis(phosphonomethyl)amino-κ*N*)methylphosphonato-κ*O*(2-)](*trans-R,R*-cyclohexane-1,2-diamine-$\kappa^2$*N*,*N*')platinum(II) compound being the most active one in this series.

These findings are in good agreement with structure-activity relationships of analogous osteotropic complexes with BPMAA as ligand (Table 1) /12/. They are also in accord with the *in vitro* /13,14/ and *in vivo* /15,16/ antitumor activity of (cyclohexane-1,2-diamine)oxalatoplatinum(II) isomers (among which the *trans-R,R* form, oxaliplatin, is known to be the most active) as well as with other (cyclohexane-1,2-diamine)platinum(II) and platinum(IV) complexes.

**Table 2**

Cytotoxic activity of [(bis(phosphonomethyl)amino-κ*N*)acetato-κ*O*(2-)]platinum(II) complexes **1-3** in the ovarian cancer cell line CH1 ($IC_{50}$ values in μM, exposure for 96 hours, MTT assay) in comparison to cisplatin, carboplatin, and oxaliplatin.

| compound | $IC_{50}$ value [μM] |
|---|---|
| cisplatin | 0.15 |
| oxaliplatin | 0.27 |
| carboplatin | 2.5 |
| **3** | 11.8 |
| **2** | 35.6 |
| **1** | 84.3 |

Coordination of the bone-seeking ATMP leaving group resulted in a marked increase of cytotoxic activity. Complexes **1-3** display an *in vitro* antitumor activity in the ovarian carcinoma cell line CH1 which is about 1.5 to 2 fold higher in comparison to their BPMAA analogues. The decrease in $IC_{50}$ values is explainable by an increased reactivity of the complexes due to the coordinated phosphonato residue in case of ATMP as ligand, whereas platinum(II) complexes with BPMAA ligands (coordinated carboxylato residue) seem to be more stable.

### 4. CONCLUSIONS

In order to explore structure-activity relationships, bone-seeking (cyclohexane-1,2-diamine)platinum(II) complexes with ATMP as ligand have been synthesized and tested for their cytotoxicity in the cisplatin-sensitive ovarian carcinoma cell line CH1. A marked improvement of *in vitro* antitumor potency could be observed in comparison to analogous BPMAA complexes.

Whether the change of the phosphonic acid ligand has a positive impact on the *in vivo* anticancer activity as well as on general toxicity and whether the osteotropic properties are influenced by selection of the ligand (BPMAA versus ATMP) cannot be judged by *in vitro* investigations and must be clarified in appropriate animal experiments.

### ACKNOWLEDGEMENTS

The support of the FWF (Fonds zur Foerderung der wissenschaftlichen Forschung), Faustus Forschungs Compagnie Translational Cancer Research GmbH and COST (European Co-operation in the Field of Scientific and Technical Research) is gratefully acknowledged.

### REFERENCES

1 B. Rosenberg, L. VanCamp and T. Krigas, *Nature*, **205**, 698-699 (1965).

2 B. Rosenberg, L. VanCamp, J. E. Trosko and V. H. Mansour, *Nature*, **222**, 385-386 (1969).

3. B. Rosenberg, *Interdiscip.Sci.Rev.*,3, 134-147 (1978).

4. B. Lippert (Ed.). *Cisplatin: Chemistry and Biochemistry of a Leading Anticancer Drug*; Verlag Helvetica Chimica Acta, Zürich (Switzerland) and Wiley-VCH, Weinheim (Germany), (1999).

5. P. J. O'Dwyer, J. P. Stevenson and S. W. Johnson, *Drugs*, **59(Suppl. 4)**, 19-27 (2000).

6. P. J. O'Dwyer, J. P. Stevenson and S. W. Johnson, In *Cisplatin: Chemistry and Biochemistry of a Leading Anticancer Drug*; B. Lippert (Ed.) Verlag Helvetica Chimica Acta, Zürich (Switzerland) and WILEY-VCH, Weinheim (Germany), pp 31-69 (1999).

7. J. Graham, M. Muhsin and P. Kirkpatrick, *Nature Rev. Drug Discov.*, **3**, 11-12 (2004).

8. M. A. Jakupec, M. Galanski and B. K. Keppler, *Rev. Physiol. Biochem. Pharmacol.*, **146**, 1-53 (2003).

9. M. Galanski, S. Slaby and B. K. Keppler, In *Relevance of Tumor Models for Anticancer Drug Development;* H. H. Fiebig, A. M. Burger (Eds.) In *Contributions to Oncology*, W. Queisser, W. Scheithauer (Eds.) Karger, **54**, pp 435-438 (1999).

10. S. Slaby, M. Galanski, S. W. Metzger and B. K. Keppler, In *Relevance of Tumor Models for Anticancer Drug Development*; H. H. Fiebig, A. M. Burger (Eds.) In *Contributions to Oncology*, W. Queisser, W. Scheithauer (Eds.) Karger, **54**, pp 201-205 (1999).

11. M. Galanski, V. B. Arion, M. A. Jakupec and B. K. Keppler, *Curr. Pharm. Design*, **9**, 2078-2089 (2003).

12. M. Galanski, S. Slaby, M. A. Jakupec and B. K. Keppler, *J. Med. Chem.*, **46**, 4946-4951 (2003).

13. L. Pendyala, Y. Kidani, R. Perez, J. Wilkes, R. J. Bernacki and P. J. Creaven, *Cancer Lett.*, **97**, 177-184 (1995).

14. Y. Kido, A. R. Khokhar, S. Al-Baker and Z. H. Siddik, *Cancer Res.*, **53**, 4567-4572 (1993).

15. M. Noji, K. Okamoto and Y. Kidani, *J. Med. Chem.*, **24**, 508-515 (1981).

16. Z. H. Siddik, S. Al-Baker, G. Thai and A. R. Khokhar, *J. Cancer Res. Clin. Oncol.*, **120**, 409-414 (1994).

